# A Comprehensive Review of the Effects of Diabetes Mellitus on the Gastrointestinal System

**DOI:** 10.7759/cureus.77845

**Published:** 2025-01-22

**Authors:** Koushal Sattaru, Mansi Thipani Madhu, Janmejay Kumar Singh, Venkataramana Kandi, Aastha Gupta, Jayashankar CA, Ojas Balaji, Nidhishri Sridhar, Vennela Talla

**Affiliations:** 1 Internal Medicine, Vydehi Institute of Medical Sciences and Research Centre, Bengaluru, IND; 2 Medicine, Vydehi Institute of Medical Sciences and Research Centre, Bengaluru, IND; 3 Medicine, Teerthanker Mahaveer Medical College and Research Centre, Moradabad, IND; 4 Clinical Microbiology, Prathima Institute of Medical Sciences, Karimnagar, IND; 5 Medicine, Maulana Azad Medical College, Delhi, IND; 6 General Medicine, Vydehi Institute of Medical Sciences and Research Centre, Bengaluru, IND

**Keywords:** diabetes mellitus, gastrointestinal tract (git), management strategies, non-alcoholic fatty liver disease (nafld), pathophysiology

## Abstract

Diabetes mellitus (DM) is a worldwide epidemic, making it a major non-communicable disease of public health concern. DM is a chronic disease affecting various organs of the body, leading to increased morbidity and frequently causing patients to seek medical care. Patients with DM often suffer from gastrointestinal disturbances, indicating the involvement of the gastrointestinal system (GIS). Common effects on the gastrointestinal tract (GIT) include esophageal dysmotility, gastroesophageal reflux disease (GERD), non-alcoholic fatty liver disease (NAFLD), glycogenic hepatopathy, gastroparesis, and enteropathy. Despite the high rates of GIT complications associated with diabetes, they are often under-recognized by physicians, leading to suboptimal treatment and a poor quality of life for patients. This article reviews the GIT manifestations of DM from the esophagus to the anal canal, including their pathophysiology and current management strategies.

## Introduction and background

Diabetes mellitus (DM) is a chronic metabolic disease characterized by persistent hyperglycemia, due to inadequate insulin production or compromised insulin action. There are two main types of DM: type 1 diabetes mellitus (T1DM) and type 2 diabetes mellitus (T2DM). Gestational diabetes occurs transiently in pregnant women. T1DM, which is hereditary and insulin-dependent, generally occurs early in life and is also known as juvenile diabetes [1-3]. T2DM typically manifests in individuals over 50 years old, but it has increasingly been observed in those under 50, likely due to sedentary lifestyles and poor nutritional habits.

According to the WHO, the global burden of diabetes has grown from 200 million people (approximately 7% of adults over 18 years old) in 1990 to more than 800 million (approximately 14% of adults over 18 years old) in 2022. The increase in incidence has been particularly significant in low- and middle-income countries (LMICs), where factors such as lack of awareness, access to healthcare, and adherence to medication contribute to the heightened diabetes burden [4, 5].

DM is a chronic disease, and uncontrolled DM affects various body systems, including the gastrointestinal system (GIS), cardiovascular, and CNSs. Therefore, it is crucial for physicians treating DM patients to carefully assess the complications of the disease. This contributes to efficient patient management and improved quality of life (QoL) [6].

DM substantially impacts the GIS, wherein patients experience complex interactions related to disease pathogenesis, highlighting a wide range of consequences that significantly affect patients' QoL [7].

Diabetic gastroparesis is an important concern regarding esophageal and stomach disorders associated with DM. It is characterized by delayed stomach emptying and can cause symptoms such as bloating, vomiting, and nausea. This disorder highlights the complicated interplay between neurological, hormonal, and vascular components, representing a broader range of diabetic gastroenteropathy. It also emphasizes how DM affects GI motility in diverse ways [7].

Physicians treating and managing DM patients need to expand their knowledge and implement clinical practices to lessen the negative impacts of DM on patient well-being by understanding the interactions between DM and GI health.

This review comprehensively discusses the consequences of DM on the GIS and explores the underlying pathophysiology, clinical symptoms, and therapeutic techniques for improved patient management.

## Review

Effects of DM on the esophagus

The effects of DM on the esophagus have not been extensively investigated. However, the prevalence of gastroparesis is higher among DM patients, with these patients generally complaining of heartburn, noted in 25-41% of DM cases [8]. DM induces various mechano-physiological effects on the esophagus, such as decreased lower esophageal sphincter tone and reduced amplitude and frequency of esophageal peristaltic movements. These effects are primarily linked to autonomic dysfunction seen in patients with long-standing DM [9]. Studies have shown that the frequency of esophageal peristaltic movements, and hence esophageal motility disorders and gastroesophageal reflux disease (GERD), worsen with the duration of DM [10]. DM has been identified as an independent risk factor for Barrett's esophagus [11] and is also a risk factor for the development of esophageal adenocarcinoma and the progression of Barrett’s esophagus to cancer [12].

DM patients with GERD are treated with proton pump inhibitors (PPIs). Those with unresolved symptoms require further evaluation using ambulatory lower esophageal pH monitoring with a wireless capsule. Additionally, a transnasal catheter with a wireless capsule is more beneficial for the patient. Although upper gastrointestinal endoscopy is not usually recommended for GERD patients, it is preferred for those demonstrating warning symptoms such as dysphagia and who are at high risk of developing Barrett's esophagus.

Patients with esophageal dysmotility often have coexisting GERD and can present with dysphagia. Such patients are evaluated using esophageal manometry, which may show features like peristaltic contractions with an amplitude <30 mmHg, simultaneous contractions <30 mmHg, and failed/absent peristalsis. High-resolution manometry and impedance manometry are recent advances that have improved the diagnosis of these disorders [13].

Treatment strategies for GERD and esophageal dysmotility include PPIs and prokinetics such as metoclopramide and erythromycin. However, minimal evidence-based options are available to treat esophageal disorders in DM patients [14].

**Figure 1:**
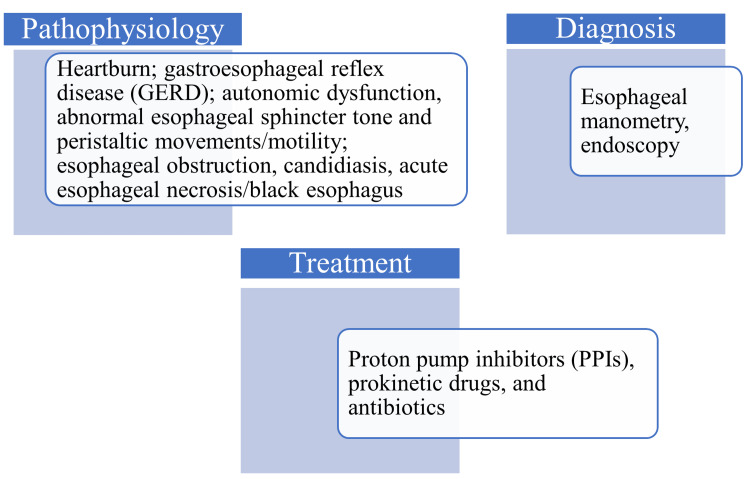
Pathophysiology, diagnosis, and management of esophageal disorders in diabetes mellitus patients. Image credit: Venkataramana Kandi.

Chronic DM resulted in esophageal obstruction in a 72-year-old patient. This patient suffered from DM-related neuropathic complications predisposing to esphageal candidiasis [15]. In diabetic ketoacidosis, patients can develop black esophagus, also known as acute esophageal necrosis or Gurvits syndrome, identified through endoscopy and attributed to necrosis due to esophageal ischemic injury, alcoholism, cardiovascular comorbidities (hypertension), and gastroesophageal acid reflux [16, 17]. Predisposing factors for black esophagus also include kidney disease, thromboembolism, malignancy, and other debilitating conditions. This condition has a poor prognosis, accounting for 30% of deaths among affected individuals (Figure 1) [18].

Effects of DM on the stomach

Diabetic gastroparesis in DM patients results in delayed gastric/stomach emptying without evidence of mechanical obstruction. Gastroparesis in DM patients presents as postprandial fullness, nausea, vomiting, and abdominal discomfort [19]. According to the available evidence, women are more likely than men to develop gastroparesis. Furthermore, significant underdiagnosis and insufficient documentation affect patient management [20].

The enteric nervous system, hormone regulation, and gastrointestinal motility are intricately entwined in the pathophysiology of diabetic gastroparesis. Gastric motility problems may be influenced by vagal dysfunction and hyperglycemia, in addition to the major role played by autonomic neuropathy. The clinical picture is further complicated by the interaction between blood glucose levels and stomach emptying [19].

The clinical presentation differs with T1DM patients, who experience symptoms earlier than those with T2DM. Differentiating between disorders such as gastric outlet obstruction and functional dyspepsia is necessary for an accurate diagnosis, requiring several diagnostic techniques, such as the urea breath test for *Helicobacter pylori* (*H. pylori*) infection and scintigraphy. Scintigraphy is a radionuclide imaging technique that allows physicians to visualize the insides of the organs and diagnose and assess the disease prognosis.

Pharmacotherapy, dietary changes, and glucose control are the mainstays of patient management. Metoclopramide is a prokinetic drug used to treat symptoms, although it can lead to side effects. Erythromycin is suggested to treat infectious etiologies. Ghrelin receptor agonists (ulimorelin) are among the novel treatments being researched to improve stomach motility [21].

**Figure 2:**
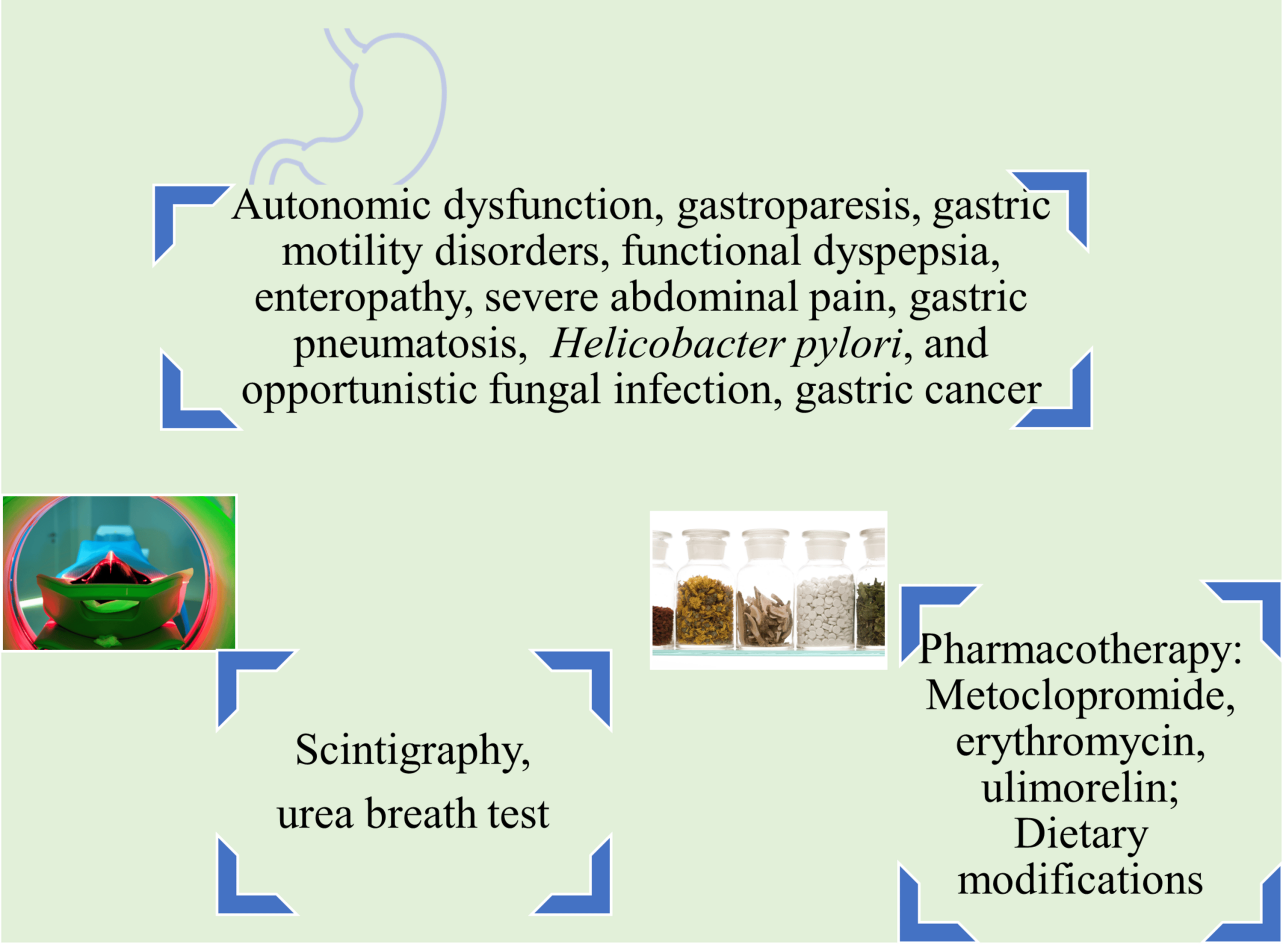
Pathophysiology, diagnosis, and treatment of stomach disorders in diabetes mellitus patients. Image credit: Venkataramana Kandi.

Persistent severe abdominal pain with recurrence was identified in a patient suffering from T1DM. This was attributed to uncontrolled blood glucose and ketoacidosis [22]. T2DM patients with hyperglycemia are predisposed to opportunistic fungal infections, as evidenced by the occurrence of mucormycosis of the gastric mucosa [23]. Although the evidence available to associate DM with gastric cancer (GC) is limited, factors contributing to the development of GC among DM patients may include obesity, hyperlipidemia, hyperinsulinemia, chronic H. pylori infections, and anti-diabetic drugs [24, 25]. A 29-year-old T1DM patient with hyperglycemia and ketoacidosis developed gastric pneumatosis, a condition characterized by the accumulation of gas within the gastric wall. This condition is generally associated with emphysematous gastritis (Figure 2) [26].

Effects of DM on the liver

The liver plays a central role in gluconeogenesis, glycogenesis, and insulin metabolism. Diabetic patients face a significantly higher risk of chronic liver disease, including non-alcoholic fatty liver disease (NAFLD), cirrhosis, and hepatocellular carcinoma (HCC) [27, 28].

NAFLD is characterized by lipid accumulation in hepatocytes despite no alcohol consumption. It is divided into two types: simple hepatic steatosis and nonalcoholic steatohepatitis (NASH). NASH, which is more severe, can progress to cirrhosis. Insulin resistance is a major contributor, leading to increased lipolysis and fat accumulation in the liver, causing inflammation and cell damage [29, 30].

DM affects individuals aged 40-60 years who are overweight or obese, and most patients remain asymptomatic. It is often diagnosed incidentally during routine checks, often showing mild liver enzyme elevations [27, 30, 31].

Imaging or biopsy is required to confirm NASH while ruling out other liver diseases. Various imaging methods include ultrasound, CT, and MRI, with biopsy as the gold standard [32]. Non-invasive biomarkers, such as cytokeratin 18 and the NAFLD fibrosis score, are promising for assessing liver damage [33-35].

Weight loss and lifestyle modifications are crucial for treating NAFLD [36-38]. Insulin sensitizers, such as metformin and pioglitazone, show varying effectiveness in improving liver health [32, 39]. Other options include antioxidants and potential treatments like glucagon-like peptide-1 (GLP-1) analogs and bariatric surgery [37, 40].

NAFLD occurs primarily in poorly controlled T1DM, characterized by glycogen overload in the liver. Symptoms may include elevated liver enzymes and abdominal pain; management primarily involves improved glycemic control [41, 42].

**Figure 3:**
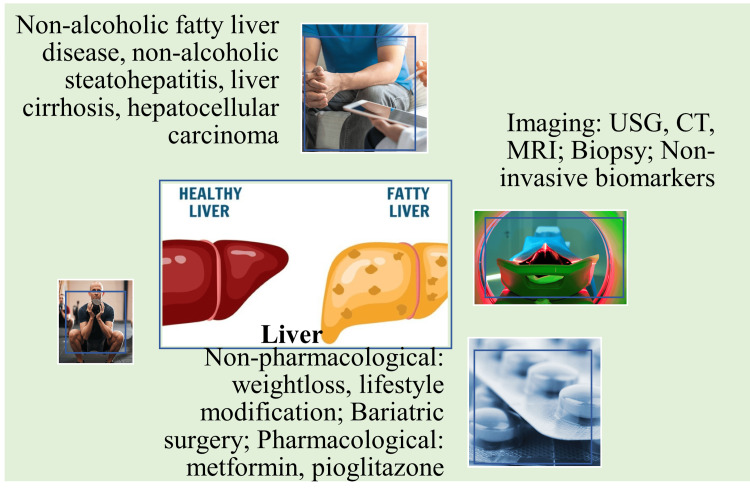
Pathophysiology, diagnosis, and treatment of liver disorders in diabetes mellitus patients. Image credit: Venkataramana Kandi.

An 18-year-old T1DM patient presented with hyperglycemia, diabetic ketoacidosis, and abdominal distension. Physical examination revealed hepatomegaly without evidence of NAFLD, steatosis, fibrosis, or portal inflammation. Hepatomegaly, in this case, could have been caused by abnormal accumulation of glycogen in the liver cells, known as glycogenic hepatopathy [43, 44]. Available research also suggests a potential relationship between DM and liver disease. People with DM are predisposed to chronic liver disease, liver cirrhosis, and HCC. HCC is more common among DM patients with comorbidities like hepatitis C and hepatitis B viral infections (Figure 3) [45].

Effects of DM on intestines

Acute hyperglycemia significantly impacts postprandial small intestinal motility, potentially causing diabetes by reducing duodenal and jejunal pressure waves and retarding duodenal-cecal transit [46, 47]. In T1DM, patients suffer from small intestinal bacterial overgrowth (SIBO). However, glucose absorption in individuals with controlled blood glucose is similar to that of healthy controls, potentially due to duodenal motility [47].

One serious side effect of T2DM affecting the small intestines is chronic diarrhea. Drugs such as metformin, which impair bile salt absorption in the ileum, cause it. Additionally, artificial sweeteners affect ileal absorption by an osmotic mechanism (primarily observed with sorbitol ingestion larger than 20 g). Alternatively, they are affected by intestinal flora, which produces short-chain fatty acids and hydrogen that cause diarrhea [48, 49].

Changes in the vagus nerve, including segmental demyelination, axonal degeneration, and reduced motor and sensory ganglia, are believed to affect the autonomic nerves of the colon [50]. Colonic tissue shows signs of myenteric neuronal loss and evidence of elevated oxidative stress [51].

Colon remodeling may reduce muscular contraction, lower compliance, and thicken the colonic wall. This is due to advanced glycation end products (AGEs) and upsurging collagen production. These changes can cause neuropathic and myopathic effects on the colon, altering motility, microbiota, and sensory and motor responses. The effects can range from mild to severe, with gastrointestinal symptoms impacting the colon's health [52].

The most frequent symptoms associated with the intestine are constipation, diarrhea, abdominal pain, and bloating. In an extensive review by Concepción et al., chronic constipation (25%) is more common than chronic diarrhea (5%) in a group of diabetic individuals. The National Health and Nutrition Examination Survey (NHANES) found that 25% of diabetics had gastrointestinal symptoms. This study reported that although chronic diarrhea was more prevalent in diabetics than non-diabetics (11.2% vs 6.0%, P < 0.0001), chronic constipation was also found to be more common in diabetics as compared to non-diabetics (4.6% vs 11.2%; p = 0.126). Furthermore, diabetic patients with chronic diarrhea tended to take more hypoglycemic medicines, particularly metformin, whereas diabetics with chronic constipation had impaired kidney function [48].

Diarrhea normally lasts more than 6 weeks; it is watery, painless, and non-bloody, and its appearance varies with the duration of DM. It begins with a regular stool frequency or even constipation, followed by a rapid increase in volume and frequency. Two of the most typical symptoms of diabetic diarrhea are nocturnal diarrhea and fecal incontinence [32].

Small intestine manometry (measures GIT smooth muscle pressure) provides information about contractile activity, but its applications are limited. Scintigraphy measures small intestinal transit, although its diagnostic value is unclear [51]. Recent technologies such as SmartPill (a wireless ingestible capsule having sensors that monitor pressure, pH, transit time, and temperature of the GIT) and 3D-Transit (an ingestible electromagnetic capsule with detector and sensors integrated into software for analysis and visualization of GIT motility and transit), require more evaluation before clinical usage. The aspiration and culture of jejunal fluid is the diagnostic standard because intestinal bacterial overgrowth is one of the main causes of gastrointestinal symptoms. Endoscopy must be used, and there is a significant risk of external contamination and false-negative results. Breath tests lack sufficient sensitivity but might be helpful [53-56].

Management involves assessing hydration status and electrolyte imbalances, aiming for good glycemic control, and managing diet. If initial measures fail, drug therapy with opioid-group antidiarrheals may be considered, but caution is advised due to their toxicity.

Antibiotic therapy is recommended for SIBO, with rifaximin (a bacteriostatic agent) being the best research-based intervention. In 33% to 99% of patients, it reduces symptoms, lowers the chance of antibiotic resistance, and targets the gastrointestinal tract (GIT) [57]. Somatostatin analogs like octreotide and lanreotide also improve symptoms by inhibiting water secretion, increasing absorptive capacity, and suppressing the release of hormones with gastrointestinal effects. Nonetheless, there is no particular medication for diabetic people with persistent diarrhea. Prokinetics target slow transit, while opiates target rapid transit [40, 49, 58].

Constipation is a common complaint in people with T2DM, and good hydration, high-fiber meals, and regular physical activities are recommended. Clinical trials show that consuming natural psyllium (10 g twice a day) or flaxseed (10 g twice a day) can reduce symptoms and improve glycemic control [59]. Treatment focuses on managing symptoms through a diet that softens stool and laxatives that increase intestinal transit [60]. The study suggests using osmotic laxatives like polyethylene glycol and stimulant laxatives like bisacodyl or picosulfate for diabetes-related constipation. Lubiprostone, a chloride channel activator, can increase colon secretion, reduce transit time, and increase spontaneous bowel movements [61, 62].

**Figure 4:**
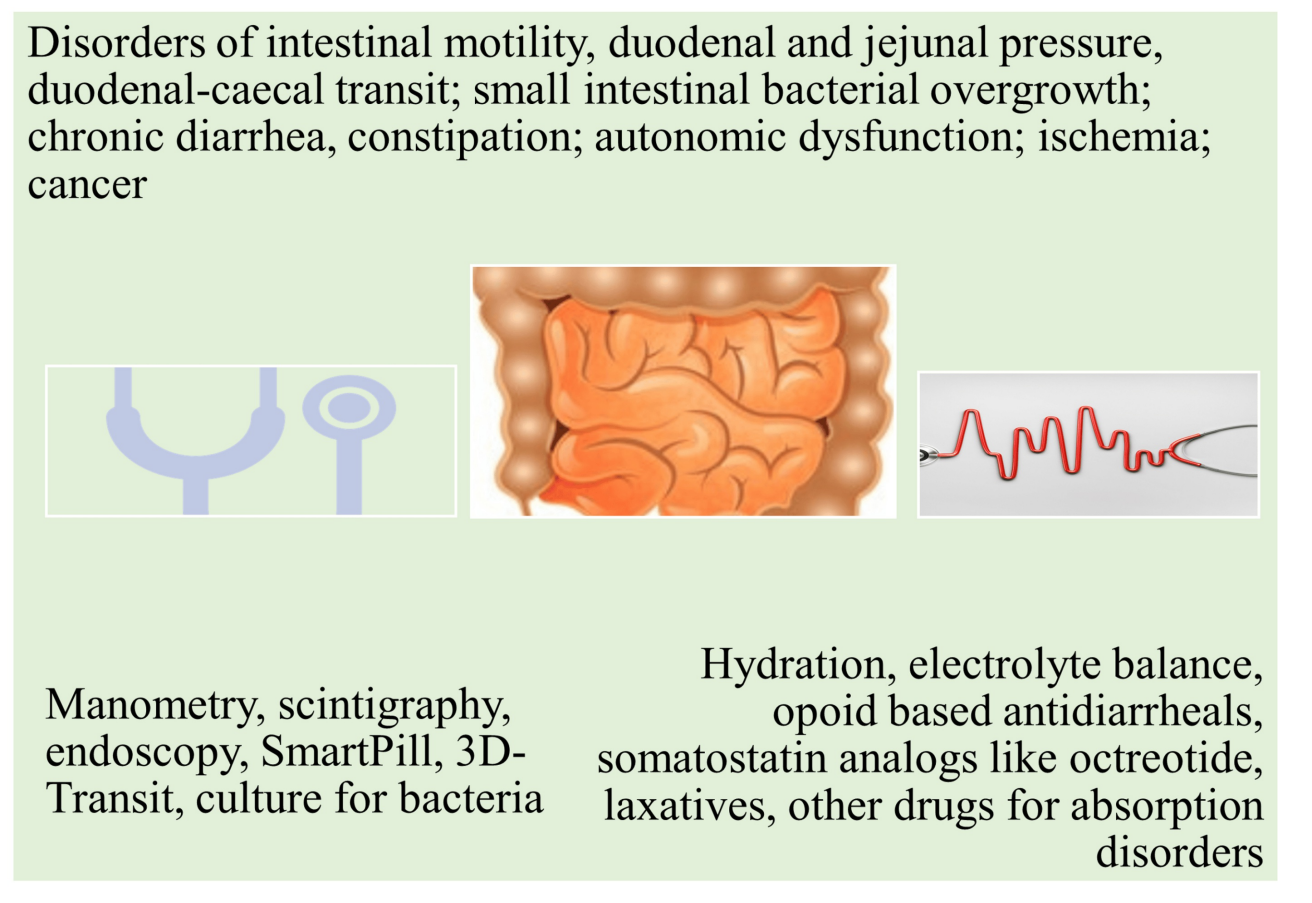
Pathophysiology, diagnosis, and treatment of intestinal disorders in diabetes mellitus patients. Image credit: Venkataramana Kandi.

Evidence suggests that chronic mesenteric ischemia (CMI) results in bowel infarction, which in turn causes intestinal hypoperfusion leading to severe abdominal pain among T2DM patients [63]. A study among T1DM patients assessed the duodenal mucosal microbial flora and the inflammatory components. The results of this case-control study revealed a disproportionate duodenal microflora with raised inflammatory markers (C-C motif chemokine ligand-CCL13, CCL19, CCL22; C-C chemokine receptor type 2-CCR2; cyclooxygenase-2 (COX2); interleukin-4 receptor (IL4R); cluster of differentiation 68 (CD68); pentraxin 3 (PTX3); tumor necrosis factor alpha (TNFα); vascular endothelial growth factor A (VGEFA)) among cases compared to the control group [64]. The gut microbial flora has been linked to the pathophysiology of DM since microbial metabolites can influence glucose metabolism. However, further extensive studies are required to substantiate the association between intestinal bacteria and DM [65]. Studies have indicated potential pathophysiological changes in the small and large intestines of people with DM. There was evidence of histomorphological changes potentially induced by DM, including effects on the mucosa, submucosa, muscle wall, and muscle, nerve, and interstitial cells of Cajal (ICC). Other effects noted in the intestines of individuals with DM include sensory and motor changes in the intestine and colon. These changes can result in compromised intestinal motility, dysbiosis, diarrhea, constipation, intussusception, and cancer [52, 66]. Intestinal ischemia in a T1DM patient with ketoacidosis reaffirms the role of uncontrolled blood glucose on intestinal pathology (Figure 4) [67].

Effects of DM on the rectum and anal canal

Patients with long-term DM have an elevated risk of fecal incontinence and substantially decreased function of both the anal sphincters and the rectum. These findings could be explained by the higher prevalence of microangiopathy, as well as autonomic and peripheral neuropathy, in this group of diabetic patients [68].

This is consistent with observations of reduced squeezing pressure and external anal sphincter (EAS) dysfunction. Lower squeezing activity in the anal canal may be associated with motor peripheral neuropathy of the pudendal nerves and S3-S4 nerve roots, which innervate the EAS and puborectalis muscle [69, 70]. The number of ICC in diabetic individuals with gastrointestinal symptoms declines, contributing to reduced internal anal sphincter (IAS) tone [71]. The rectum receives autonomic innervation from sympathetic and parasympathetic nerves. Rectal sensation is mediated by parasympathetic fibers that travel from S2 to S4, and reduced rectal sensibility in diabetic individuals may be a further indication of diabetic autonomic neuropathy (DAN) [72].

Fecal incontinence is more common in chronic DM patients due to microvascular complications. IAS tone and contraction pressures are reduced, with prevalence ranging from 7%-15%. Anal itching is a typical sign of nerve loss in diabetics, and itching sensations can be caused by injured nerve fibers in the skin surrounding the anus. These nerves provide sensory information and are especially vulnerable to high blood sugar, making them prone to neuropathy, a type of nerve damage common in diabetics [73].

Anorectal manometry and intestinal transit tests can diagnose constipation disorders, whereas serological studies are recommended for diabetic individuals due to the increased prevalence of celiac disease in diabetic patients.

To manage fecal incontinence, it is important to identify the underlying cause of diarrhea. Good glycemic control can benefit patients [58, 62]. Neuromodulatory electrical stimulation (NES) of the sacral nerve is an emerging treatment technique for fecal incontinence and possible sensitivity in the anal canal. NES utilizes electrical interfaces to alter the activity of the nervous system. However, it has not been specifically investigated in people with diabetic gastroenteropathy. Operant reconditioning of recto-sphincteric responses can treat fecal and urinary incontinence by improving the quality of voluntary contraction of the external anal sphincter [74, 75].

**Figure 5:**
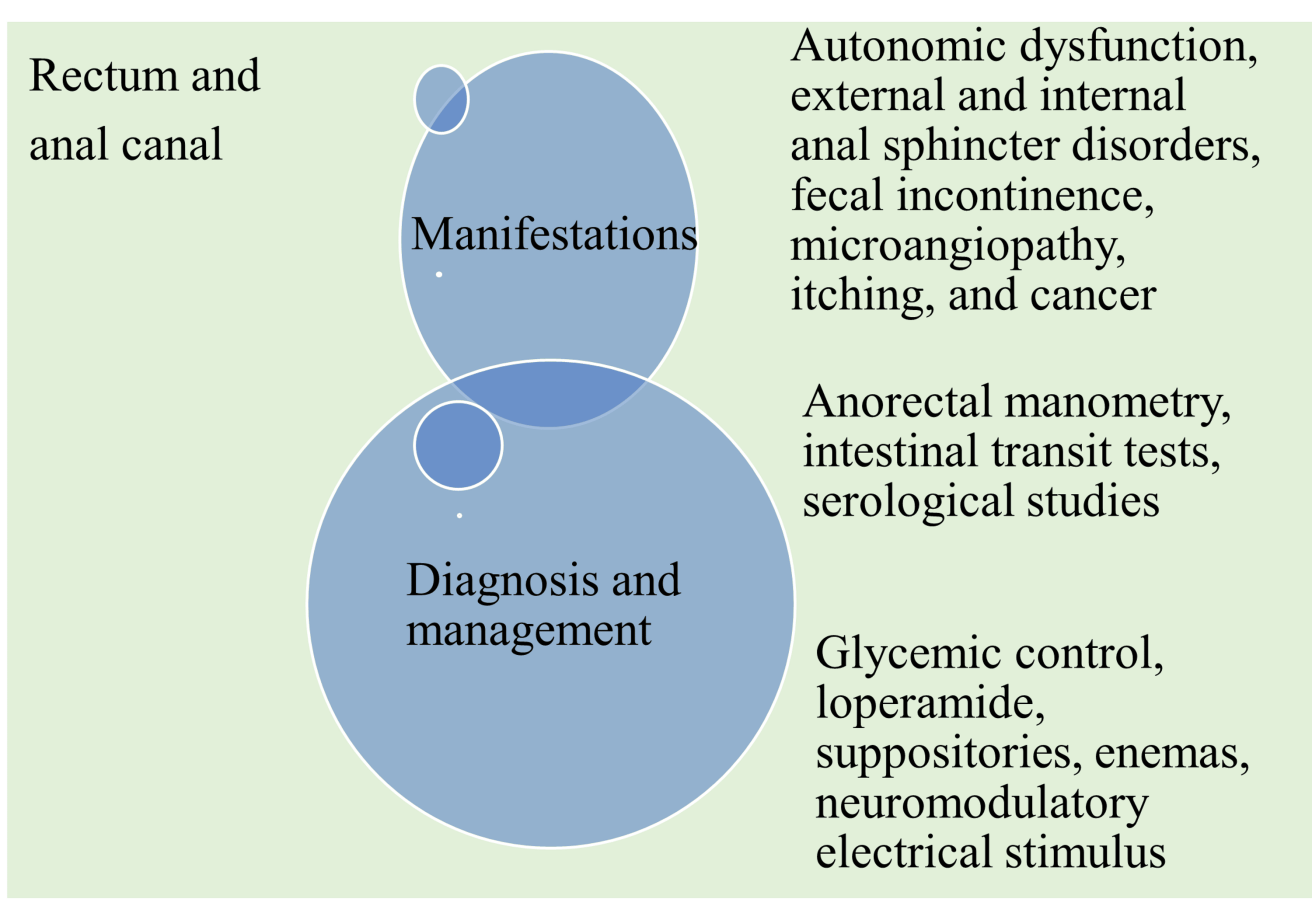
Pathophysiology, diagnosis, and management of rectum and anal canal disorders in diabetes mellitus patients. Image credit: Venkataramana Kandi.

DM causes defects in the autonomic nervous system as noted by the variability in rectal sensitivity to heat, mechanical distention, and electrical stimulus [76]. The anal sphincter pressure was found to be reduced in rats with hyperglycemia compared to nondiabetic rats. Hyperglycemic rats also demonstrated increased oxidative stress and elevated nitric oxide activities [77]. A case-control study confirmed that patients with T2DM are at a 47% increased risk of developing colorectal cancer compared to nondiabetic persons [78]. Rectal hypersensitivity was observed in longstanding and recently diagnosed T2DM patients compared to control subjects [79]. An increased risk for right-sided colon cancer (RCC) (RR = 1.35, 95% CI = 1.24-1.47) and left-sided colon cancer (LCC) (RR = 1.18, 95% CI = 1.08-1.28) with RCC being most common among DM patients was observed (Figure 5) [80].

## Conclusions

The impacts of DM on the GIS are extensive and complex. The complex interplay between DM and the GIS greatly impacts patients' health, leading to diverse clinical symptoms and outcomes. One major problem is diabetic gastroparesis, characterized by early satiety, nausea, and vomiting. These symptoms can significantly impair QoL and complicate blood sugar regulation. Autonomic neuropathy and reduced stomach motility are among the many causes of diabetic gastroparesis, highlighting the need for a multimodal treatment approach. Effective management of this illness requires glycemic control, dietary changes, and pharmaceutical treatments.

The study also highlights the significant impact of DM on liver health, particularly conditions like NAFLD and glycogenic hepatopathy. NAFLD, which can progress from mild hepatic steatosis to more severe forms, is a major concern. This is particularly relevant given that people with DM have a higher risk of dying from chronic liver diseases. Accurate diagnosis, targeted therapy, and early screening are essential. Despite the encouraging advancements in non-invasive biomarkers and imaging, liver biopsy is still required for a precise diagnosis. To address these liver-related issues, new treatments are necessary, along with a focus on weight and glycemic control.

Strict monitoring, effective management practices, and continuous research are all necessary components of a comprehensive plan to address these issues. Developing customized treatments and understanding the pathways linking DM to these systemic issues would improve patient outcomes and QoL. To manage the complex relationships between DM and its systemic effects, interdisciplinary teamwork, better patient education, and enhanced diagnostic and therapeutic approaches are needed.
